# Dr. A. J. Volck's Elaine and Repousse Work

**Published:** 1884-11

**Authors:** 


DR. VOLCK'S ELAINE AND OTHER REPOUSSE WORK.—For several years past Dr. A. J. Volck, a practicing dentist of this city, has been occupying his leisure hours with the production of a high order of repousse work. His artistic success in this line has been very remarkable. The repousse work to which he has turned his attention has not been of the kind that is seen in tea services and other silverware. What he has sought is to make it a feature in American decorative art that shall rival the best of similar work that has been done abroad. The three pieces he has completed are masterly in composition and charming in their effects. They are all in the form of shields of different shapes, and each shield, of copper, covered in two of them with a film of silver, tells in the composition of the piece a different story or an episode in history. Thus, in the Cortez shield, we have a representation of the tremendous struggle between the Aztecs and the Spaniards in front of the City of Mexico, as told in Prescott's history and known as "The Battle of the Causeway." The figures, buddied together in all the conceivable positions incidental to a conflict restricted to a small space, are not only distinctively defined in dress and national characteristics, but are full of life and action. This shield is well known to members of the Wednesday Club and their guests, for it hung for some time on the walls of the club, although it may now be seen at the art gallery of Myers & Hedian. It is a stricking piece of modelling, but does not carry with it the interest of the Arthur shield, representing the incidents of "The Passing of Arthur," from Tennyson's Idyls of the King, the central eatures of which is the bearing off of Arthur in the mystics barge by draped figures across the mere to the wonderful isle of Avilion. But even this episode in the romance of King Arthur, well as it has been wrought out by the artist, has not the surpassing interest of his last work, which tells, in a series of scenes, the touching story of "The Lily Maid of Astelot," who pined away for love of Sir Launcelot, and whose fair body, put into a barge guided by one old servant, ficated with the tide until, by some strange chance, it was arrested near in the court of King Arthur and laid in sorrowful state in the great hall, as in the painting by Hovenden, In the repousse shield by Volck, we have the salient points of this sad story. The central piece represents Elaine reclining in an antique chair, contemplating the shield of Launcelot. The skin of a wild animal lies stretched on the floor by her side, the shield is on a support in front of her, and in the background a window. Above this central figure, yet forming a part of her meditations, is seen a tournament, that knightly passage of arms where some of the constants are unhorsed, some wounded, and only one can be proclaimed victor in the fray.. On the left hand side of the central piece we have Launcelot, with his attendants, taking their departure from Astelot, and remotely seen on a balcony in the background, the figure of Elaine. On the right-hand side of the central Elaine, assisted by her brother, is alighting from her horse at the mouth of the hermit's cave, outside of which hangs the helmet of Sir Launcelot, and within which Launcelot lies wounded. The last scene represents the body of Elaine, lying, draped, in a barge, floating upon the river, guided by an ancient servant of Astelot. All these tender passages in the story are brought out clearly, and with rare skill, in flats or high relief on the shield. By these gradations in relief some of the figures are brought fully into prominence, while in respect to others in the background there is the illusion of distance. The sharpness with which the figures are outlined, as well as the artistic excellence of the composition, has elicited high praise from connoisseurs, while the contrasted textures and the remarkable aerial effects have been spoken of by persons accustomed to repousse work as something beyond what has heretofore been known in this country, and presenting features which are unusual in the very best work of the kind.

